# Targeting *CCNE1* amplified ovarian and endometrial cancers by combined inhibition of PKMYT1 and ATR

**DOI:** 10.21203/rs.3.rs-3854682/v1

**Published:** 2024-02-16

**Authors:** Haineng Xu, Erin George, David Gallo, Sergey Medvedev, Xiaolei Wang, Rosie Kryczka, Marc L. Hyer, Jimmy Fourtounis, Rino Stocco, Elia Aguado-Fraile, Adam Petrone, Shou Yun Yin, Ariya Shiwram, Matthew Anderson, Hyoung Kim, Fang Liu, C. Gary Marshall, Fiona Simpkins

**Affiliations:** 1 Penn Ovarian Cancer Research Center, Perelman School of Medicine, University of Pennsylvania, Philadelphia, PA; 2 Department of Obstetrics and Gynecology, Division of Gynecologic Oncology, Hospital of the University of Pennsylvania, Philadelphia, PA; 3 Repare Therapeutics, Inc., 7171 Frederick-Banting, Ville St-Laurent, QC, Canada.; 4 Repare Therapeutics, Cambridge, MA, USA

**Keywords:** PKMYT1, ATR, CCNE1 amplification

## Abstract

Ovarian cancers (OVCAs) and endometrial cancers (EMCAs) with *CCNE1-*amplification are often resistant to standard of care treatment and represent an unmet clinical need. Previously, synthetic-lethal screening identified loss of the CDK1 regulator, PKMYT1, as synthetically lethal with *CCNE1*-amplification. We hypothesized that *CCNE1*-amplification associated replication stress will be more effectively targeted by combining the PKMYT1 inhibitor, lunresertib (RP-6306), with the ATR inhibitor, camonsertib (RP-3500/RG6526). Low dose combination RP-6306 with RP-3500 synergistically increased cytotoxicity more in *CCNE1* amplified compared to non-amplified cells. Combination treatment produced durable antitumor activity and increased survival in *CCNE1* amplified patient-derived and cell line-derived xenografts. Mechanistically, low doses of RP-6306 with RP-3500 increase CDK1 activation more so than monotherapy, triggering rapid and robust induction of premature mitosis, DNA damage and apoptosis in a *CCNE1*-dependent manner. These findings suggest that targeting CDK1 activity by combining RP-6306 with RP-3500 is a novel therapeutic approach to treat *CCNE1*-amplifed OVCAs and EMCAs.

## Introduction

Despite significant progress in the last twenty years in ovarian cancer (OVCA) treatment and improved mortality rates^1^, this disease remains the most lethal gynecologic malignancy^2^. Such progress has particularly favored patients with germline or somatic *BRCA1/2* pathogenic mutations or those with tumors exhibiting homologous recombination (HR) deficiency^3,4^. On the other hand, patients with HR proficient tumors, particularly those with *CCNE1* gene amplification, exhibit *de novo* or rapid emergence of chemotherapy resistance and poor survival^5,6^. Endometrial cancer subtypes share molecular profiles with HGSOC such as *CCNE1* amplification and *TP53* mutations. There is an increased incidence in high-risk EMCA histologic subtypes such as uterine serous carcinoma and carcinosarcoma^7^, and these subtypes are among the cancers with highest incidence of *CCNE1* gene amplification^8^. Further, mortality rates for endometrial cancer (EMCA) are increasing and the two-fold higher risk of death from ovarian cancer compared to endometrial cancer in the early 1990s has virtually been eliminated by oppositional mortality trends^1^. To date, there are no FDA-approved drugs for common *CCNE1* amplified cancers, such as OVCA and EMCA, despite preclinical data showing *CCNE1* amplification is targetable therapeutically^9–12^. Given this unmet clinical need, we sought to identify a treatment strategy that targets critical survival pathways for *CCNE1*-amplified dependent gynecological cancers.

Cyclin E1 binds and activates CDK2, a key regulatory element that promotes initiation of DNA replication and G1/S cell cycle progression^13,14^. *CCNE1* amplification and cyclin E1 overexpression prematurely activate CDK2 and initiation of DNA replication before cells have time to sufficiently license pre-replication complexes at origins^15^. Unscheduled origin firing lead to replication stress and ensuing DNA damage and genome instability^9,11,16^. Cyclin E-CDK2 complexes directly activate the MYBL2-MuvB-FOXM1 (MMB) transcriptional network causing early activation of the G2/M transcriptional network and accumulation of cytoplasmic cyclin B in S-phase^17^. To slow the onset of mitosis and allow time to compete faithful genome duplication, *CCNE1* amplified cells activate the S and G2/M cell cycle checkpoints to suppress cell cycle transitions, allowing time to complete DNA replication^11^. We reasoned that increased dependance on S and G2/M checkpoints for survival in *CCNE1* amplified cells represents a therapeutic vulnerability which is the focus of this study.

Membrane-associated tyrosine- and threonine-specific Cdc2-inhibitory kinase (PKMYT1) and WEE1 kinase are each critical G2/M checkpoint regulators that inhibit cell cycle progression by catalyzing inhibitory phosphorylation of CDKs^18–21^. WEE1 restricts both CDK1 and CDK2 activity by phosphorylating Tyr15, while PKMYT1 selectively restricts CDK1 activity by phosphorylating Thr14^22^. WEE1 is localized to the nucleus, while PKMYT1 is tethered to the cytoplasmic face of the ER/Golgi, where it physically interacts with cyclin B^21,23^. PKMYT1 and WEE1 inhibitors (PKMYT1i and WEE1i, respectively) are each reported to show preclinical efficacy as monotherapy in *CCNE1*-amplified and cyclin E overexpressing models by activating CDK1, which forces cells into mitosis prematurely with under replicated and damaged DNA^17,24,25^.

Replication stress caused by *CCNE1* amplification also activates the ataxia telangiectasia and Rad3-related (ATR) kinase to promote the DNA damage response (DDR), stabilizing DNA replication forks from collapse into DNA double strand breaks and preventing dormant origin activation^11^. ATR also activates CHK1 that limits mitotic progression by phosphorylating and inhibiting the CDC25 family phosphatases required for dephosphorylation and activation of CDK1^26^. *CCNE1* amplification also sensitizes cells to ATR inhibition^27^.

Previous studies indicate that targeting S and G2/M checkpoint kinases selectively kill *CCNE1*-amplified and overexpressing cells. WEE1 inhibitors (WEE1i) are reported to show preclinical efficacy as monotherapy in *CCNE1*-amplified and overexpressing models.^17,24,25^. In certain contexts, ATR inhibitors (ATRi) are also reported to show cytotoxic activity in *CCNE1* overexpressing models with high levels of replication stress^27^. We have previously reported that low doses of WEE1i and ATRi synergize in *CCNE1*-amplified OVCA and EMCA preclinical models by increasing replication fork collapse and DNA damage^11^. Intriguingly, a recent CRISPR-Cas9 genome-wide screen using an isogenic pair of cell lines that stably overexpress cyclin E from a *CCNE1-2A-GFP* fusion integrated into the genome of RPE1-hTERT *TP53*^*−/−*^ Cas9 cells identified PKMYT1 as a top hit for synthetic lethality in the *CCNE1* overexpressing cells. This study further showed that PKMYT1 inhibition (PKMYT1i) alone induces cell death by forcing S-phase into mitosis with under-replicated DNA resulting in mitotic catastrophe^17^. Given *CCNE1*-amplified cells rely on the G2/M checkpoint to attenuate lethal levels of replication stress induced DNA damage by upregulating the ATR axis for repair of DNA damage^24^, we hypothesized that dual inhibition of PKMYT1 and ATR (PKMYT1i-ATRi) will further enhance CDK1 activation and cytotoxicity especially towards *CCNE1* amplified or overexpressing tumor cells allowing lower dosing strategies and potentially alleviate toxicity.

There is currently one PKMYT1i and several ATRi in clinical development. Lunresertib (RP-6306), is a novel, first in class, selective and orally bioavailable PKMYT1i^28^. RP-6306 is currently in clinical development as monotherapy or in combination with chemotherapy or targeted therapies (e.g. camonsertib) for treatment of patients with solid tumors harboring *CCNE1* amplification or *FBXW7/PPP2R1A* inactivating mutations (NCT04855656, NCT06107868, NCT05147272)^29^. There are currently seven ATR inhibitors in clinical development (RP-3500,AZD6738, BAY1895344, ATRN119, M1774, ART0380, IMP9064; NCT04497116, NCT01955668, NCT03188965, NCT04905914, NCT04170153, NCT04657068, NCT05269316). Camonsertib (RP-3500) is potent, selective and orally bioavailable ATR inhibitor currently in clinical development as monotherapy and in combination with other targeted therapies including PARPi for treatment of patients with solid tumors harboring selected DDR alterations, including *BRCA1/2* or *ATM* inactivating mutations (NCT04972110, NCT05405309)^30,31^.

In this study, we report that combining RP-6306 with RP-3500 (PKMYT1i-ATRi) increases CCNE1 level-dependent cytotoxicity in a large panel of OVCA and EMCA cell lines. The increased cytotoxicity translated to significantly improved activity and overall survival in mice bearing *CCNE1*-amplified cell lines and patient-derived xenografts. Increased cytotoxicity and activity was attributed to synergistic CDK1 activation in S-phase of *CCNE1* overexpressing or amplified cells triggering unscheduled mitosis before completion of DNA replication leading to lethal levels of DNA damage and increased apoptosis. We find that *CCNE1* amplification is a robust biomarker predictive of sensitivity towards low doses of PKMYT1i-ATRi, with limited effects observed in *CCNE1*^*LOW*^ or *BRCA1/ATM* mutated cells. Taken together our studies support the clinical development of PKMYT1i-ATRi combinations for treatment of tumors with *CCNE1* amplification and/or overexpression.

## Results

### Combination PKMYT1i-ATRi is synergistic in *CCNE1* amplified OVCA and EMCA cells.

**Figure 1.**
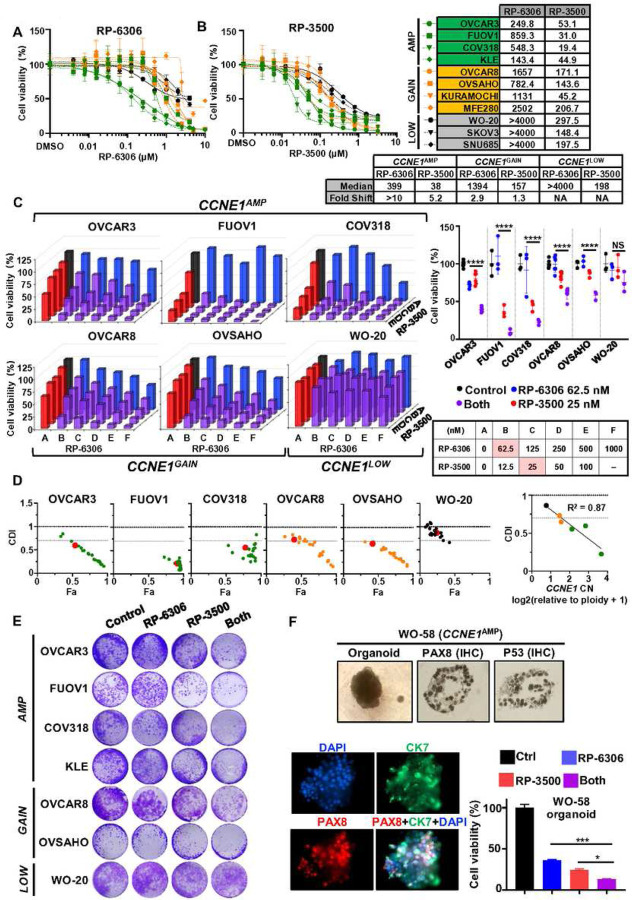
Combination PKMYT1i-ATRi is synergistic in CCNE1 amplified OVCA and EMCA cells. **(A-B)** Detection of cells response to monotherapy of PKMYT1i, RP6306 **(A)** and ATRi, RP-3500 **(B)** with MTT assay. *CCNE1*^AMP^ in blue, *CCNE1*^GAIN^ in orange, and *CCNE1*^LOW^ in black. **(C)** Cell viability analysis of the indicated *CCNE1* amplified, gain, and low/neutral cell lines after treatment at indicated doses. Monotherapy for PKMYT1i, RP6306, is highlighted in blue and ATRi, RP3500, is highlighted in red. Combinations are highlighted in purple. Assays were normalized by doubling time such that cells doubled at least twice. *n*=3; Mean was presented. Most synergistic dose found was compared (right) and highlighted in red in the table (bottom right). Growth inhibition relative to DMSO control of indicated cells after treatment with pink highlighted doses. n=3; Mean ± S.D. **(D)** Coefficient of drug interaction (CDI) relative to fraction affected (Fa) plot of the indicated cell lines. *CCNE1*^AMP^ in Green, *CCNE1*^GAIN^ in orange, and *CCNE1*^LOW^ in black. CDI<1 synergy with CDI<0.7 significant synergy, CDI=1 additive, CDI>1 antagonistic. Plot of CDI versus *CCNE1* copy number at indicated dose highlighted in pink (right). **(E)** Colony formation Analysis of PKMYT1i-ATRi combination in *CCNE1* amplified OVCA and EMCA cells with RP-6306 (31.3nM), RP-3500 (6.25nM) or combination for 10 days. **(F)** Cell viability detection of PKMYT1i-ATRi combination on *CCNE1* amplified HGSOC organoids. Organoids were developed from *CCNE1* amplified, BRCA1 mutant HGSOC WO-58 and characterized with ovarian cancer marker PAX8 and epithelial marker CK7 by immunofluorescence (IF) and immunohistochemistry (IHC). P53 expression was detected by IHC. WO-58 organoids were treated with RP-6306 (250nM), RP-3500 (50nM), or both for 10 days and measured with CCK8 assay. n=3; Mean + SD. ****P<0.0001, ***P<0.001, *P<0.05, NS: P>0.05.

To investigate the dependence of PKMYT1i on CCNE1 level in OVCA (OVCAR3, FUOV1, COV318, OVCAR8, OVSAHO, Kuramochi, WO-20, SKOV3) and EMCA (KLE, MFE280, SNU685) cells, RP-6306 efficacy in cancer cells with *CCNE1* amplification (*CCNE1*^AMP^), *CCNE1* copy gain (*CCNE1*^GAIN^), or *CCNE1* neutral (*CCNE1*^LOW^) was investigated. RP-6306 was most active in *CCNE1*^AMP^ cells, with reduced activity in *CCNE1*^GAIN^ cells and very limited effect in *CCNE1*^LOW^ cells (Fig. 1A, Fig. S1A). Since PKMYT1 inhibition can force cells into unscheduled mitosis by preventing CDK1 phosphorylation, we hypothesized that this effect could be enhanced by activation of CDC25 via inhibition of ATR. We therefore tested the ATR inhibitor (ATRi), RP-3500, in combination studies with RP-6306. High CCNE1 expression levels did not significantly enrich for RP-3500 single-agent activity as robustly as for RP-6306 (Fig. 1B, Fig. S1A). Combination of RP-6306 with RP-3500 showed a much stronger inhibitory effect than the respective monotherapy treatments, with significant synergistic effect by coefficient of drug interaction (CDI) in *CCNE1*^AMP^ cells, less so in *CCNE1*^GAIN^ cells, and very limited to no effect in *CCNE1*^LOW^ cells (Fig. 1C). *CCNE1* copy number (CN) correlated with CDI (R^2^ = 0.87, Fig. 1D). Fraction affected (Fa) cells and CDI calculation clearly illustrates that the synergistic effect of the combination decreases viability in a *CCNE1*-dependent manner by lower CDI values with higher fractions of cells affected (Fig. 1D). Colony formation assays yielded similar results, with the combination of RP-6306 and RP-3500 significantly inhibiting colony formation in *CCNE1*^AMP^ cells, somewhat less effectively in *CCNE1*^GAIN^ cells, with minimal effects in *CCNE1*^LOW^ cells (Fig. 1E, Fig. S1B). To evaluate the efficacy of these compounds in a more clinically relevant model, we established patient-derived organoids from a *BRCA1* mutant, *CCNE1*^AMP^ (CN=7) PDX model with acquired PARP inhibitor resistance, designated WO-58. This organoid model is characterized by ovarian marker PAX8 and epithelial marker CK7 (Fig. 1F). Combination of RP-6306 and RP-3500 showed better growth inhibition of WO-58 organoids than the respective monotherapies (Fig. 1F). Taken together, these results suggest that PKMYT1i-ATRi combination is more effective in OVCA and EMCA with higher level of *CCNE1* copy number.

### *CCNE1* induction augments PKMYT1-ATRi cytotoxic effects

**Figure 2.**
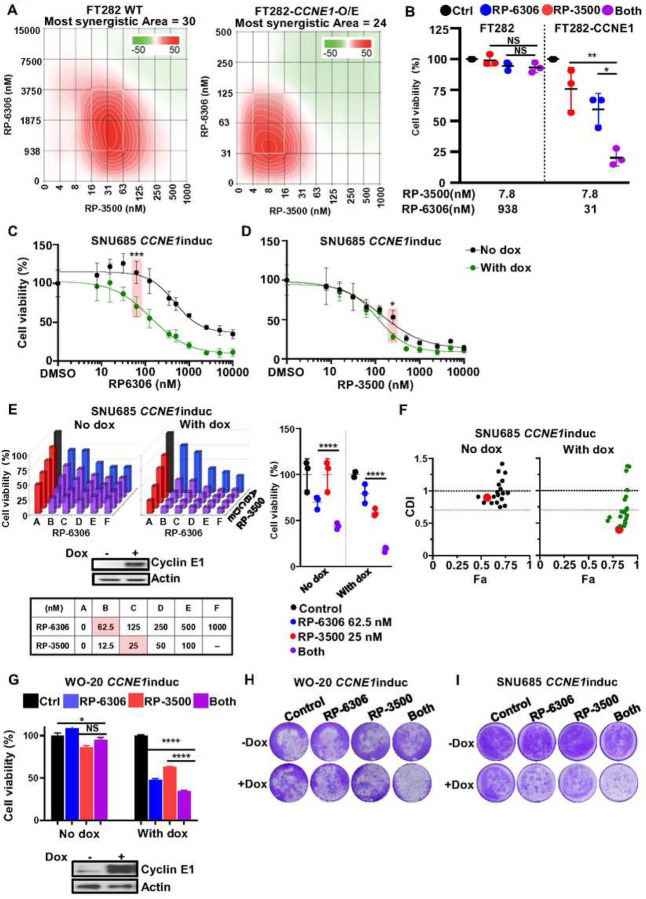
Combination PKMYT1i-ATRi is synergistic in OVCA and EMCA cells depending on CCNE1 level. **(A)** ZIP synergy scores at various dose combinations of RP-6306 and RP-3500 in FT282-hTERT *p53*^R175H^ parental (WT) and CCNE1-overexpressing (*CCNE1*-O/E) cells. Score ≥10 (red color) represents synergy, ≤−10 (green) represents antagonism. Values were obtained by analyzing mean data from 3 independent biological replicates with SynergyFinder. **(B)** Growth inhibition relative to DMSO control of parental and CCNE1-overexpressing cells after treatment with the indicated dose of RP-6306, RP-3500 or the combination of both. n=3; Mean + SD. **(C-D)** Cell viability detection of SNU685 cells in response to RP-6306 monotherapy (C) and RP-3500 monotherapy (D). n=3; Mean ± SD. Highlighted dose showing statistical difference in *CCNE1* induced SNU685 cells with or without *CCNE1* induction. **(E-F)** Cell viability analysis of the indicated SNU685 *CCNE1* inducible cells ± doxycycline lines after treatment at the indicated doses. Doxycycline: 1µg/ml. Monotherapy for PKMYT1i, RP6306, is highlighted in blue, for ATRi, RP3500, is highlighted in red, Combinations are highlighted in purple for PKMYT1i-ATRi. Assays were normalized by doubling time such that cells doubled at least twice. n=3; Mean± SD. Growth inhibition relative to DMSO control of parental and *CCNE1*-overexpressing cells after treatment with indicated doses in pink (middle). Coefficient of drug interaction (CDI) relative to fraction affected (Fa) plot of the indicated cell lines is indicated to the right of each bar graph. CDI<1 synergy with CDI<0.7 significant synergy, CDI=1 additive, CDI>1 antagonistic. Red dot corresponds to doses highlighted in pink. **(G)** Measurement of drugs combinations in WO-20 *CCNE1*^*inducible*^ cells with or without Cyclin E1 induction. n=4; Mean + SD. **(H-I)** Colony formation analysis of WO-20 *CCNE1*^*inducible*^ cells **(H)** and SNU685 *CCNE1*^*inducible*^ **(I)** in response to RP-6306 (31.3nM), RP-3500 (6.25nM) or combination for 10 days. ****P<0.0001, ***P<0.001, **P<0.01, *P<0.05, NS: P>0.05.

To further investigate whether the PKMYT1i-ATRi combination is dependent on Cyclin E1 protein level, we established and then tested drugs in immortalized fallopian tube cells and OVCA and EMCA cell lines, with and without *CCNE1* expression (FT282 with *CCNE1* overexpression, WO-20 with inducible *CCNE1* and SNU685 with inducible *CCNE1*)^11^. Strong synergy was observed in both parental and *CCNE1* overexpressing FT282 cells. However, the concentration of RP-6306 required for synergy was about 60-fold lower, and for RP-3500 about 4-fold lower, in *CCNE1* overexpressing cells (Fig. 2A). While 938 nM RP-6306 and 7.8 nM RP-3500 showed no effect in combination on parental FT282 cells, FT282-*CCNE1* cells treated with only 31 nM RP-6306 and 7.8 nM RP-3500 showed almost 80% growth inhibition (Fig. 2B). In SNU685 cells, induction of *CCNE1* increased sensitivity to RP-6306 by 4.8-fold alone, but had a limited effect on sensitivity to RP-3500 (Fig. 2C–D and Fig. S2A). The PKMYT1i-ATRi combination showed limited activity in SNU685 cells, but with *CCNE1* induction, the combination was much more effective and demonstrated synergy (Fig. 2E–F). The effect of *CCNE1* induction on enhancing combination benefit in the OVCA WO-20 cell line was similar to that observed in SNU685 (Fig. 2G). The PKMYT1i-ATRi combination also inhibited colony formation in WO-20 and SNU685 cells with *CCNE1* induction but very limited effect was observed in paired Cyclin E1 low cells (Fig 2H–I and Fig. S2B). These results demonstrated that combination inhibition of PKMYT1i-ATRi clearly relies on cyclin E1 expression level for a cytotoxic effect.

### PKMYTi-ATRi causes tumor regression in *CCNE1* amplified PDX models

To study the *in vivo* anti-tumor activity of PKMYT1i-ATRi combination, three patient-derived xenograft (PDX) models with *CCNE1* amplification were utilized, including two OVCA (WO-19, WO-77) and one EMCA (WU-115) model (Fig. S3A-B). First, tolerability and anti-tumor activity were explored using RP-6306 and RP-3500 monotherapy in NSG mice, and mice bearing WO-19 xenograft tumors. Both RP-6306 and RP-3500 were tolerated as indicated by stable body weights at their maximum tolerated doses (MTDs), of 20 mg/kg for both compounds (Fig. S4A left and bottom). Both RP-6306 and RP-3500 monotherapy yielded limited single agent anti-tumor activity in WO-19 PDX (Fig S4B), necessitating evaluation of the PKMYT1i-ATRi combination.

**Figure 3.**
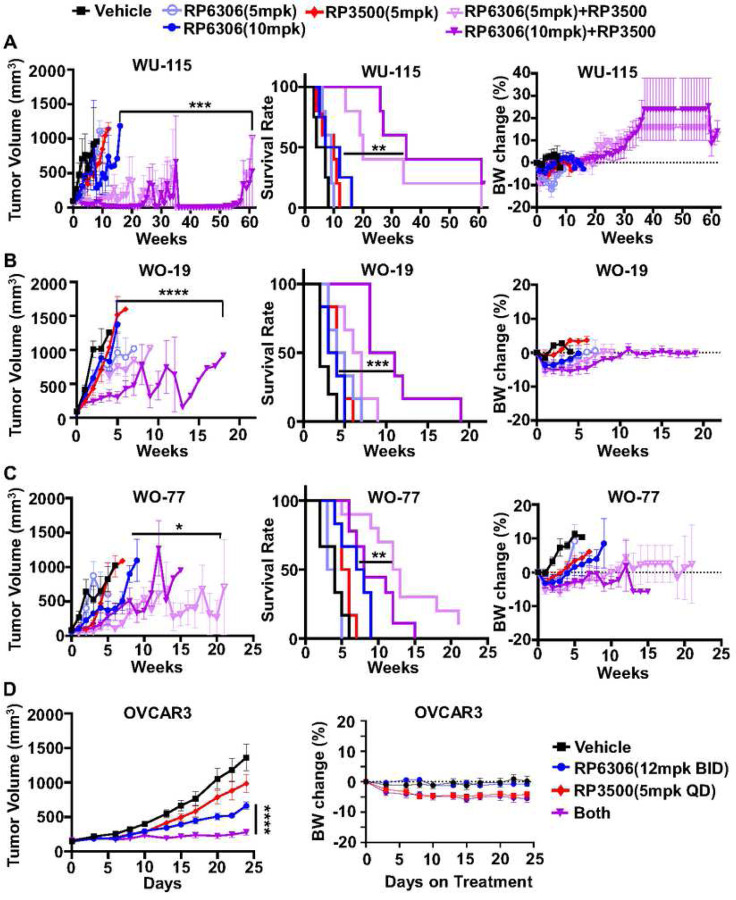
Combination of PKMYTi-ATRi synergistically suppress CCNE1 amplified OVCA and EMCA PDXs growth. (A-C) Tumor volume growth was measured weekly in (A) EMCA WU-115 (B) OVCA WO-19 and (C) OVCA WO-77 *CCNE1* amplified PDX models treated with the indicated drugs. RP-6306 was given oral BID on days 1–5 and RP-3500 was given oral QD on a 3 days on / 4 days off schedule until tumor progression (tumor volume >1000 mm^3^). Survival rate (middle panel) was analyzed at the end of each experiment. The toxicity of drugs was revealed by the mice body weights (right panel) changes. (D) Tumor growth (left) and body weight change (right) of OVCAR3 xenografts in mice treated with either RP-6306, RP-3500 or both. RP-3500 was given oral QD and RP-6306 was given oral BID, both given on a 3 day on / 4 day off schedule for 24 days (n=8). Tumor growth and percent body weight change shown is mean ± SEM. Longitudinal tumor growth was analyzed by linear mixed effects modeling with type II ANOVA and pairwise comparisons across groups. Data were analyzed for overall survival using Mantel-Cox log-rank test. Body weight shown is mean ± SEM. *p<0.05; **p<0.01; ***p<0.001; ****p<0.0001; ns, not significant.

In a tolerability study, non-tumor bearing NSG mice were treated with combination of RP-6306 and RP-3500, using multiple dose levels, in order to select doses to evaluate in an efficacy study using tumor bearing mice. There were no treatment cessations required for the mice in the combination RP-6306 at 5 mg/kg or 10 mg/kg with RP-3500 using intermittent dosing at 5 mg/kg and only two dose reductions in the combination RP-6306 at 10 mg/kg with RP-3500 at 5 mg/kg (Fig. S4A right and bottom), yielding tolerated doses to be evaluated in tumor bearing efficacy studies. Next, mouse plasma pharmacokinetic studies were performed to evaluate drug-drug interactions and overall free fraction exposure. The free plasma concentrations of RP-6306 and RP-3500 were similar when dosed as single agents or when used in combination suggesting no significant drug-drug interactions (Fig S4C), and exposures were at or above levels previously associated with meaningful target pathway engagement ^28,29^. Combination RP-6306 at 5 mg/kg or 10 mg/kg with RP-3500 (5 mg/kg) resulted in significant tumor regressions in the WU-115 model compared to both RP-6306 at 10 mg/kg (P=0.0240, P=0.0025, respectively) and RP-3500 monotherapy (P=0.0032, P=0.002, respectively, Fig. 3A and Fig. S5A). There was over a 6-fold improvement in median overall survival compared to control and an approximate 3.5-fold improvement relative to monotherapies with limited toxicity as evident by body weight (Fig. 3A). Combination PKMYT1i-ATRi also increased tumor suppression in the *CCNE1*^AMP^ OVCA WO-19 and WO-77 PDX models, which are resistant to standard-of-care chemotherapy (Fig. 3B–C, Fig. S5B and Fig. S6A)^11^. For WO-19, combination RP-6306 at 10 mg/kg with RP-3500 (5 mg/kg) significantly increased tumor suppression relative to RP-6306 at 10 mg/kg (P<0.0001) and RP-3500 (P<0.0001) monotherapy and improved median overall survival relative to monotherapy (P=0.0007, P=0.0006, respectively) with limited toxicity (Fig. 3B). For WO-77, combination RP-6306 at 5 mg/kg with RP-3500 (5 mg/kg) significantly increased tumor suppression relative to RP-6306 at 5 mg/kg (P<0.0001) and RP-3500 (P=0.0063) monotherapy and improved median overall survival relative to monotherapy (P<0.0001 and P=0.0003, respectively) with limited toxicity (Fig. 3C). We also tested combination PKMYT1i-ATRi in the *CCNE1*^AMP^ OVCAR3 cell line xenograft and found superior tumor growth inhibition (TGI) with the combination compared to RP-6306 and RP-3500 monotherapy (P<0.001) and showed limited toxicity (Fig. 3D and Fig, S6B). These data collectively demonstrate that combination inhibition of PKMYT1 and ATR significantly suppresses tumor growth and improves median overall survival in *CCNE1*^AMP^ OVCA and EMCA.

### ATRi enhances DNA damage and premature mitosis caused by PKMYT1i

**Figure 4.**
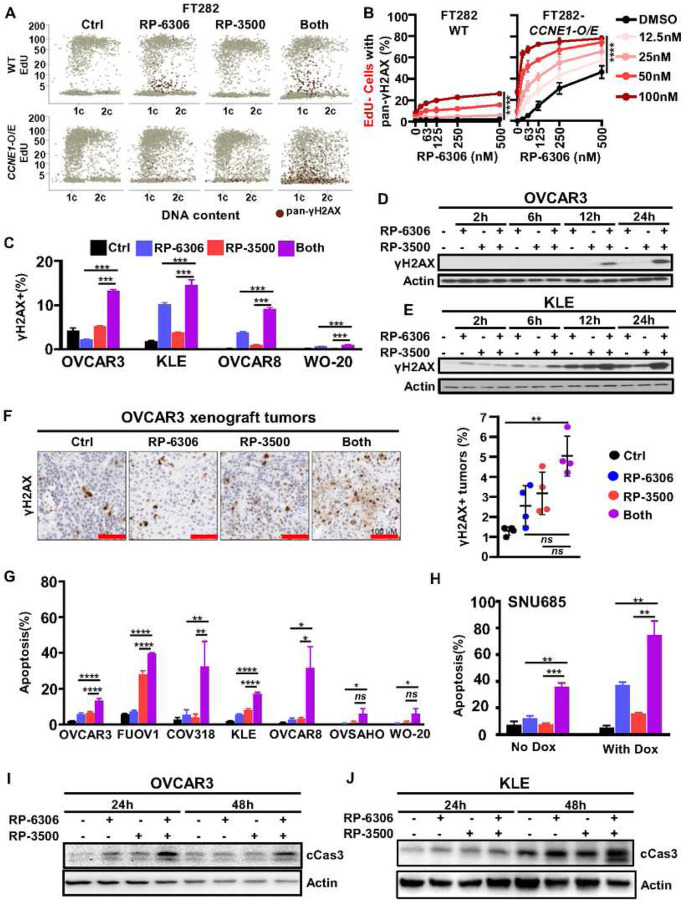
Dual inhibition of PKMYT1 and ATR induced DNA damage and cell apoptosis in CCNE1 amplified cancers. **(A)** Representative QIBC plots of γH2AX nuclear intensity, EdU incorporation and DNA content (DAPI) in FT282-hTERT *p53*^*R175H*^ parental (WT) and *CCNE1-*overexpressing (*CCNE1-*O/E) cells treated with RP-6306 (63 nM), RP-3500 (50 nM) or combination of both treated for 48 h. **(B)** QIBC quantitation of FT282-hTERT *p53*^R175H^ parental (WT, left) and *CCNE1*-overexpressing (*CCNE1*-O/E, right) EdU^−^/pan-γH2AX^+^ cell in response to the indicated RP-6306/RP-3500 combinations treated for 48 h. n=3; Mean + SD. **(C)** Detection of γH2AX^+^ cells by flow cytometry in indicated cells after treated with RP-6306 (250nM), RP-3500 (50nM), or combination of both treated for 24 h. n=3; Mean + SD. **(D-E)** Whole cell lysates of OVCAR3 (D) and KLE (E) cells were treated with RP-6306 (250nM), RP-3500 (50nM), or both for the indicated times and immunoblotted with γH2AX and Actin antibodies. Actin is loading control. **(F)** OVCAR3 tumor-bearing mice were administered RP-6306 (5 mg/kg) orally BID, RP-3500 (5 mg/kg) orally QD or combination of both for 3 days, sacrificed 2h post last treatment and tumor tissue was prepared for FFPE. Tumor tissues were stained with γH2AX antibodies (left) and the percentage of γH2AX strong positive tissue (right) present in the tumor area was quantified by HALO software. n=4; Mean + SD. Scale bar: 100 µm. **(G)** Flow cytometry quantification of apoptotic cells with Annexin V and propidium iodide (PI) staining of the indicated cells after treated with drugs RP-6306 (250nM), RP-3500 (50nM), or both for 72 hrs. n=3; Mean + SD **(H)** SNU685 CCNE1 inducible cells were treated and detected with apoptotic cells same as (G). n=3; Mean + SD. **(I-J)** Whole cell lysates of OVCAR3 (I) and KLE (J) cells were treated with RP-6306 (250nM), RP-3500 (50nM), or both for the indicated times and immunoblotted with cCas3 and Actin antibodies. Actin is loading control. ****P<0.0001, **P<0.01, *P<0.05, NS: P>0.05.

We investigated if defective cell cycle progression and DNA damage causes cell death in PKMYT1i-ATRi treated FT282 *CCNE1*-overexpressing cells. To measure cell cycle progression and DNA damage, we used quantitative imaging-based cytometry (QIBC) with 5-ethynyl-20 - deoxyuridine (EdU) incorporation to label S-phase cells, 4’,6-diamidino-2-phenylindole (DAPI) to measure DNA content and γH2AX as a marker of DNA damage (Fig. S7A). The combination of RP-6306 with RP-3500 led to a substantial increase in DNA damage in *CCNE1* overexpressing FT282 cells based on the appearance of pan-γH2AX-positive (pan-γH2AX^+^) cells (Fig. 4A–B). Most of the pan-γH2AX^+^ cells had <2C DNA content and were EdU^−^, suggesting the cells with DNA damage were unable to complete DNA replication (Fig. 4A). Consistent with a defect in DNA replication, the proportion of EdU^+^ *CCNE1*-overexpressing cells was dramatically decreased when treated with RP-6306/RP-3500 compared to RP-6306 or RP-3500 alone (Fig. S7B). Notably, the most dramatic increase of DNA damage in *CCNE1*-overexpressing cells was observed at RP-6306 and RP-3500 concentrations that have little effect as single agent or on parental FT282 cells (Fig. 4B). Combination RP-6306 with RP-3500 treatment increased γH2AX levels in *CCNE1*^AMP^ (OVCAR3, KLE and FUOV1) and *CCNE1*^GAIN^ (OVCAR8) but not *CCNE1*^LOW^ (WO-20) cells indicating that *CCNE1* amplification arising from tumors is susceptible to DNA damage induced by combination PKMYT1i-ATRi (Fig. 4C–E and Fig. S7C). We also observed increased γH2AX in OVCAR3 xenografts, suggesting that DNA damage accumulates in PKMYT1i-ATRi treated tumors (Fig. 4F). DNA damage was accompanied by apoptosis in PKMYT1i-ATRi treated *CCNE1*-amp and *CCNE1*-overexpressing cells, as demonstrated by increased Annexin V staining (Fig. 4G–H) and elevated cleaved caspase-3 (Fig. 4I–J). Importantly, high levels of apoptosis were dependent on *CCNE1* amplification (Fig. 4G) or expression (Fig. 4H). Together, these results suggest PKMYT1i-ATRi induces lethal amounts DNA damage in cells with elevated *CCNE1* copy number or cyclin E expression.

**Figure 5.**
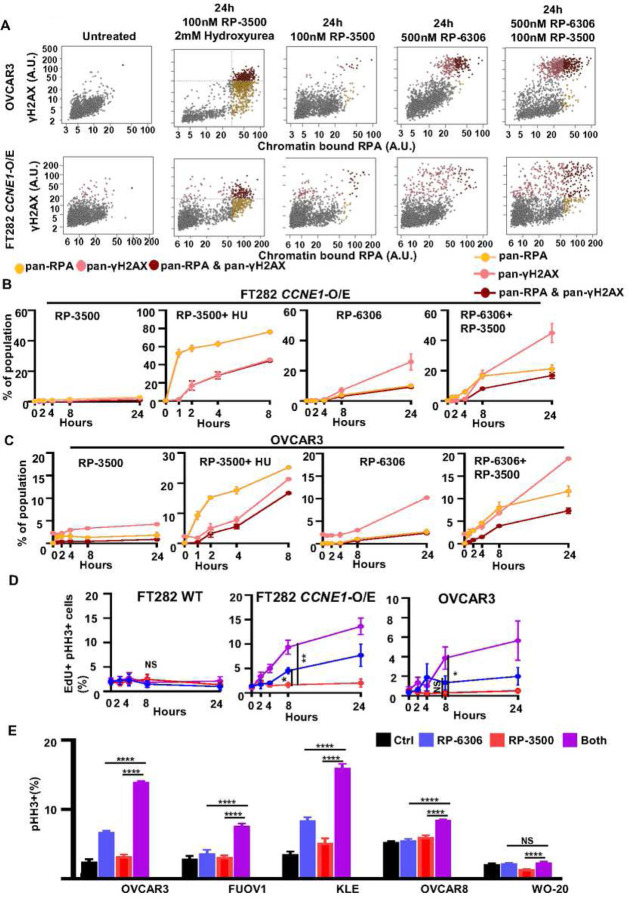
PKMYT1i-ATRi combination resulted double strand DNA in CCNE1 amplified OVCA and EMCA. **(A)** Representative γH2AX and RPA nuclear intensity QIBC plots of FT282-hTERT *p53*^*R175H*^ *CCNE1-*overexpressing (*CCNE1-*O/E) and OVCAR3 cells treated with the indicated conditions. **(B-C)** QIBC quantitation of FT282-hTERT *p53*^*R175H*^ *CCNE1-*overexpressing (*CCNE1*-O/E, B) and OVCAR3 (C) cells with percent of only pan-γH2AX^+^, only pan-RPA^+^ or both pan-γH2AX^+^/pan-RPA^+^ as a function of time after addition of RP-3500 (100 nM), RP-3500 (100 nM) and hydroxyurea (2 mM), RP-6306 (500 nM) or RP-6306 (500 nM) and RP-3500 (100 nM). (**D)** QIBC quantitation of FT282-hTERT *p53*^*R175H*^ parental (WT, left) *CCNE1-*overexpressing (*CCNE1*-O/E, middle) and OVCAR3 (right) cells with percent of EdU^+^/ pHH3^+^ as a function of time after addition of RP-6306 (250 nM), RP-3500 (100 nM) or combination of both. **(E)** Measurement pHH3^+^ cells in the indicated cells after treated with 50 nM RP-3500 (50 nM), 250nM RP-6306 (250 nM) or combination for 24 h. n=3; Mean + SD. ****P<0.0001, NS: P>0.05.

Previous studies using ATRi in combination with WEE1i or agents that induce replication stress established that irreversible levels of DNA damage arise from DNA replication defects and exhaustion of available pool of replication protein A (RPA) leading to replication catastrophe from conversion of single-stranded DNA (ssDNA) to double strand breaks (DSBs)^11,32^. We tested if the source of pan-γH2AX in PKMYT1i-ATRi treated *CCNE1*-overexpressing/*CCNE1*-amplified cells originated from replication catastrophe by simultaneously measuring chromatin-bound RPA and γH2AX using QIBC (Fig. S8A). Cells treated with RP-3500 and hydroxyurea to induce replication stress showed the characteristic replication catastrophe profile with the emergence of cells with pan-RPA preceding those with pan-γH2AX (Fig. 5A–C). In contrast, the majority of PKMYT1i and PKMYT1-ATRi treated *CCNE1*-overexpressing FT282 and OVCAR3 cells accumulated pan-γH2AX before the appearance of pan-RPA suggesting that RPA exhaustion is not causing DNA damage (Figure 5A–C). We note there is slight induction of pan-RPA^+^/γH2AX^−^ cells in ATRi treated cells at later timepoints indicating a small contribution of replication catastrophe. We conclude the predominant mechanism of pan-γH2AX induction in PKMYT1i-ATRi treated *CCNE1*-overexpressing/*CCNE1*-amplified cells do not result from replication fork breakage or replication catastrophe.

PKMYT1 inhibition in *CCNE1*-overexpressing/*CCNE1*-amplified cells activates CDK1 in S-phase triggering premature mitotic entry leading to chromosome pulverization and cell death^17^. Considering ATR inhibits cell cycle progression and CDK1 activation during S-phase^33,34^, we examined if premature mitotic entry is causing DNA damage in PKMYT1i-ATRi treated cells by measuring the proportion of EdU-positive (EdU^+^) cells marked by histone H3 Ser10 phosphorylation (H3-pS10, Fig. S8B). PKMYT1i treatment alone increased premature mitotic entry of *CCNE1*-overexpressing FT282 and OVCAR3 cells based on the emergence of histone H3 Ser10 phosphorylation (H3pS10^+^) in EdU^+^ cells (Fig. 5D). Importantly, addition of ATRi at doses that have no single agent effect increased the proportion EdU^+^/H3pS10^+^ cells. Increased H3-pS10 expression was also observed in PKMYT1i-ATRi treated *CCNE1*^AMP^ (KLE and FUOV1) cells but not in *CCNE1*^GAIN^ (OVCAR8) or *CCNE1*^LOW^ (WO-20) cells indicating a conserved mechanism-of-action in tumor-derived *CCNE1*-amplified models (Fig. 5E). These results suggest that ATR is reducing the CDK1 activation potential of RP-6306 and limiting induction of premature mitosis in *CCNE1*-overexpressing/*CCNE1-*amplified cells.

### RP-3500 cooperates with RP-6306 to activate CDK1 in S-phase

**Figure 6.**
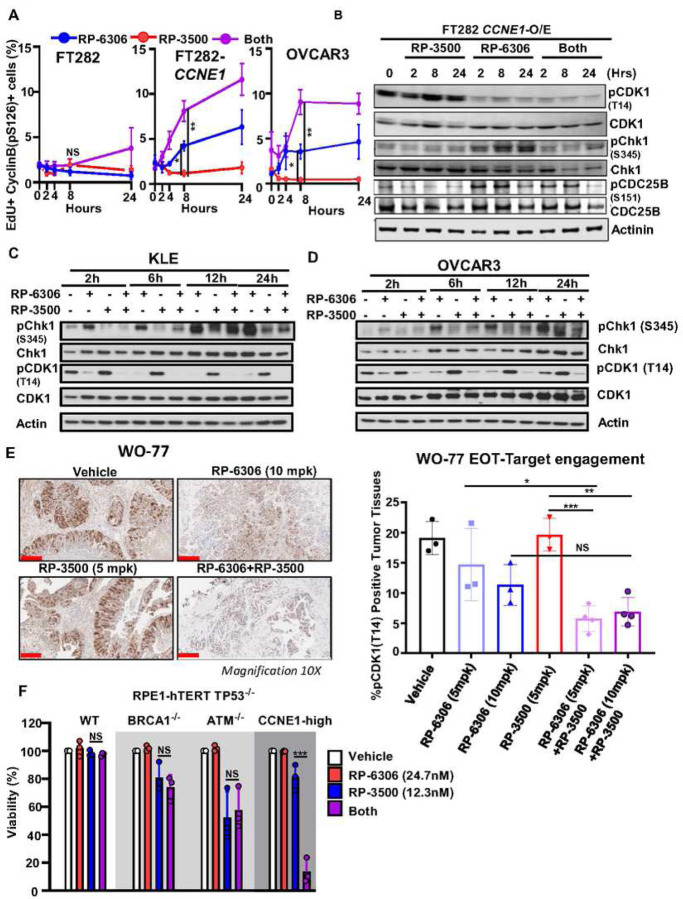
PKMYT1i-ATRi leads to premature mitosis in CCNE1 amplified OVCA and EMCA. **(A)** QIBC quantitation of FT282-hTERT *p53*^R175H^ parental (WT, left) *CCNE1*-overexpressing (*CCNE1*-O/E, middle) and OVCAR3 (right) cells with percent of EdU^+^/cyclin B-pS126^+^ as a function of time after addition of RP-6306 (250 nM), RP-3500 (100 nM) or combination of both. **(B)** Whole cell lysates of FT282-hTERT *p53*^R175H^ CCNE1-overexpressing (*CCNE1*-O/E) cells treated with RP-3500 (100nM), RP-6306 (250nM) or both for the indicated times were immunoblotted with CDK1, CDK1-pT14, CHK1, CHK1-pS345, CDC25B, CDC25B-pS151 and Actinin specific antibodies Actinin is used as loading control. **(C-D)** Whole cell lysates of KLE (C) and OVCAR3 (D) cells treated with RP-6306 (250nM), RP-3500 (50nM), or both for the indicated times were immunoblotted with CDK1, CDK1-pT14, CHK1, CHK1-pS345 and Actin specific antibodies **(E)** Tumor tissue from WO-77 tumor-bearing mice from figure 3C was prepared for FFPE at end of treatment. Tumor tissues were stained with CDK1-pT14 antibodies (left) and the percentage of CDK1-pT14 positive tissue (right) present in the tumor area was quantified by HALO software. n=4; Mean ± SD (). Detection of pCDK1-T14 level in WO-77 with RP-6306, RP-3500 or combination of both treatments by IHC. The intensity of pCDK1-T14 positive signals were quantified by HALO software. Scale bar: 200 µm. **(F)** Growth inhibition relative to DMSO control of RPE1-hTERT *TP53*^*−/−*^ parental, *BRCA1*^*−/−*^, *ATM*^*−/−*^ and CCNE1-overexpressing (*CCNE1-2A-GFP*) cells after treatment with the indicated dose of RP-6306, RP-3500 or the combination of both. n=3; Mean + SD.. ***P<0.001, **P<0.01, *P<0.05, NS: P>0.05.

We postulated that cyclin B-CDK1 activation in S-phase precedes premature mitotic entry in *CCNE1*-overexpressing/*CCNE1-*amplified cells treated with PKMYT1i-ATRi. At the onset of prophase, cyclin B-CDK1 complexes are rapidly activated and imported into the nucleus marked by CDK1 autophosphorylation of cyclin B on Ser126^35,36^ (cyclin B-pS126^+^). In PKMYT1i treated *CCNE1*-overexpressing FT282 and OVCAR3 cells, cyclin B-pS126 accumulated in the nucleus of EdU^+^ cells (Fig. 6A and Fig. S9A) and addition of ATRi increased the proportion of EdU^+^/cyclin B-pS126^+^ cells. Only a mild increase in EdU^+^/cyclin B-pS126^+^ cells was observed at later time points in FT282 parental cells, indicating that high CCNE1 expression underpins robust and premature CDK1 activation by PKMYT1i-ATRi.

To investigate how PKMYT1 and ATR inhibition are cooperating to activate CDK1, we measured levels of the CDK1 inhibitory phosphorylation at Thr14 (CDK1-pT14) in cell lines and tumor xenografts. As expected, PKMYT1i treatment reduced CDK1-pT14 levels in *CCNE1*-overexpressing/*CCNE1-*amplified cell lines (Fig. 6B–D and Fig. S9B) and *CCNE1*-amp OVCAR3 and WO-77 xenografts (Fig. 6E, Fig. S9C). Remarkably, combination of PKMYT1i-ATRi facilitated a greater reduction in CDK1-pT14 levels compared to PKMYT1i alone in cell lines (Fig. 6B–D and Fig. S9B) and WO-77 xenografts (Fig. 6E), suggesting that ATRi is bolstering PKMYT1i-dependent dephosphorylation and activation of CDK1. In response to DNA damage, ATR activates CHK1 which halts G2/M progression by catalyzing inhibitory phosphorylation of CDC25B/C phosphatases^37^. CDC25B and CDC25C phosphatases act in a sequential and coordinated manner to activate CDK1 by dephosphorylating CDK1. We reasoned that the DNA damage generated by PKMYT1i treatment leads to ATR-CHK1 activation and CDC25B inhibition, which is relieved by ATRi. We investigated this by monitoring activating phosphorylation of Ser345 on CHK1 (CHK1-pS345) and inhibitory phosphorylation of Ser151 on CDC25B (CDC25B-pS151). In *CCNE1*-overexpressing FT282 cells both CHK1-pS345 and CDC25B-pS151 levels increased upon PKMYT1i treatment and were partially suppressed by addition of ATRi. (Fig. 6B and Fig. S9B). CHK1-pS345 and CDK1-pT14 levels were also reduced by ATRi addition to PKMYT1i in *CCNE1*-amp OVCAR3 and KLE cells (Fig. 6C–D). Together, these results suggest that addition of ATRi to PKMYT1i permits rapid and robust CDK1 activation by stimulating CDK1 dephosphorylation.

Our current studies identified a strong synergistic interaction between low doses of PKMYT1i-ATRi in *CCNE1*-overexpressing/*CCNE1-*amplified preclinical models. We compared the sensitivity of PKMYT1-ATRi combination in an isogenic panel of RPE1-hTERT *TP53*^*−/−*^ parental, *ATM*^*−/−*^*, BRCA1*^*−/−*^ *and CCNE1-*overexpressing cells (Fig. S9D). The concentration of PKMYT1i that attained the highest synergy was lower in *CCNE1*-overexpressing compared to parental, *ATM*^*−/−*^ and *BRCA1*^*−/−*^ cells, indicating the synthetic lethal window of RP-6306 in *CCNE1*-overexpressing cells was exacerbated by addition of ATRi (Fig. 6F and Fig. S9E). For example, combining 24.7 nM PKMYT1i with 12.3 nn ATRi was cytotoxic in *CCNE1*-overexpressing but not in the parental, *ATM*^*−/−*^ or *BRCA1*^*−/−*^ counterparts (Fig. 6F). These results suggest that CCNE1-amplification, rather than ATM or BRCA1/2 inactivation, may associate with tumor sensitivity to PKMYT1i-ATRi combinations.

**Figure 7.**
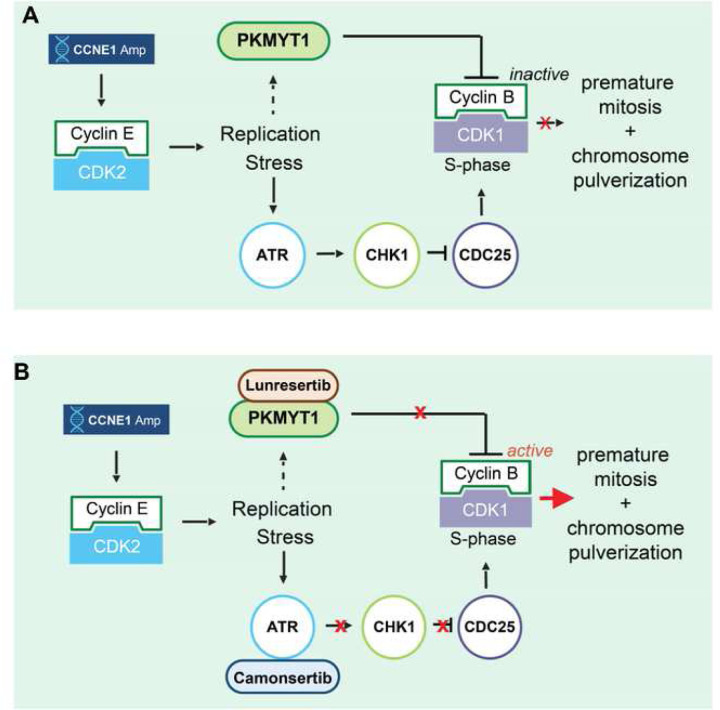
Schematic model of targeting CCNE1 amplified cancers with dual inhibition of PKMYT1 and ATR. **(A-B)** Model of synergy between PKMYT1 and ATR inhibition in *CCNE1*-amplified or overexpressing cells. **(A)** CCNE1 amplification or overexpression in cells causes replication stress and S-phase elongation. To delay induction of mitosis until DNA replication is complete CDK1 activity is inhibited by increased PKMYT1 inhibitory phosphorylation on CDK1-Thr-14 and decreased CDC25 phosphatase activity via ATR-CHK1 signaling. **(B)** Inhibition of PKMYT1 (lunresertib) reduces CDK1 The14 phosphorylation and inhibition of ATR (camonsertib) increases CDC25 activity resulting in rapid and robust S-phase CDK1 activation and premature mitosis with synergistic induction of DNA damage and cell death.

In summary, we propose a model where the synergistic cytotoxicity of PKMYT1i-ATRi originates from the ability of ATR to help PKMYT1 keep CDK1 activity low during S-phase in *CCNE1*-overexpressing cells (Fig. 7). PKMYT1i causes DNA damage that activates ATR-CHK1 and represses CDC25 phosphatases limiting CDK1 activation potential. Combined PKMYT1i-ATRi increases CDC25 phosphatase activity allowing deeper dephosphorylation of CDK1-pT14 which rapidly drives S-phase cells into mitosis resulting catastrophic DNA damage and cell death (Fig. 7).

## Discussion

*CCNE1* is commonly amplified in gynecological cancers such as OVCA and EMCA, and effective treatments exploiting this genomic alteration are currently lacking^5,6,38^. Given that this subset of cancers are typically resistant to standard-of-care platinum chemotherapy and associated with poor overall survival, we sought to address this clinical unmet need by identifying a treatment strategy that targets critical survival pathways for *CCNE1*-driven cancers^5,6^. We previously identified PKMYT1 as a new synthetic lethal target for *CCNE1* amplified cells using a genome-wide CRISPR screen approach^17,28^. PKMYT1 inhibition with RP-6306 is a selective and potent CDK1 activator, leading to mitotic catastrophe especially in CCNE1 amplified models^17^. Because emergence of resistance to monotherapy for oncogene-addicted cancers is essentially universal^39^, a combination strategy was investigated. Further, combination strategies that exploit genomic vulnerabilities can permit utilization of drug concentrations lower than required to be active as monotherapy, thereby decreasing toxicity^40^. Considering *CCNE1* amplification causes replication stress that activates the DNA replication fork stabilizer and regulator of G2/M checkpoint kinase ATR, we sought to further improve anti-tumor efficacy by combining PKMYT1 inhibition (RP-6306) with ATR inhibition (RP-3500).

Here we demonstrate that combined inhibition of PKMYT1 and ATR is synergistic in *CCNE1* amplified OVCA and EMCA cells compared to non-amplified models and effects are CCNE1 copy number dependent (Figure 1C, D, Figure 2C, E, F). We demonstrate a significant increase in anti-tumor activity and overall survival compared to monotherapy alone in *CCNE1* amplified OVCA and EMCA PDX models using a low dosing strategy justifying further evaluation in the clinic (Figure 3). We observed that combination PKMYT1 with ATR inhibition led to defective DNA replication (Figure S7B-C) and induced lethal amounts of DNA damage in cells with elevated *CCNE1* copy number or cyclin E expression as evidence by increased γH2AX (Figure 4B–D) and Annexin V (Figure 4G, H) as well as cleaved caspase 3 (Figure 4I, J). Notably the most dramatic increase in DNA damage and cytotoxicity in *CCNE1*- overexpressing/amplified cells was observed at PKMYT1i and ATRi concentrations that have little effect as single agent or in immortalized fallopian tube cells (FT282) without *CCNE1* overexpression (Figure 2 and Figure 4), thus suggesting clinical tolerability. Importantly, our work demonstrated that mutation of *BRCA1* or *ATM*, the clinical biomarkers for ATRi or ATRi-PARPi sensitivity, show little to no synergy with low doses of PKMYT1i-ATRi. Considering the synthetic lethal relationship between *CCNE1* -*BRCA1* and the near mutually exclusivity of *CCNE1* amplification and *BRCA1* mutations in tumors^41^, results from our work support inclusion of tumors with *CCNE1* amplification and exclusion of those with homologous recombination or ATM deficiencies for combination PKMYT1 with ATR inhibition.

Other effective combination studies targeting *CCNE1* amplification and or expression have been identified preclinically and clinically. Preclinically, we recently showed that CCNE1 amplification is a biomarker of response to combination WEE1 with ATR inhibition (WEE1i-ATRi)^11^. Mechanistically the WEE1i-ATRi combination anti-tumor effects differ from PKMYT1i-ATRi combination. We previously showed that combination WEE1i-ATRi treatment results in irreversible levels of DNA damage that arise from DNA replication defects and exhaustion of the available pool of RPA, ultimately leading to replication catastrophe from conversion of single strand DNA to double-strand breaks (DSBs)^11,32^. In this study, we demonstrated that the major mechanism of pan-γH2AX induction in PKMYT1i-ATRi treated cells does not result from replication fork breakage or replication catastrophe, as evidenced by accumulation of pan-γH2AX that occurred before the appearance of pan-RPA (Figure 5A–C, and Figure S8A). We provide evidence that ATRi bolsters the ability of PKMYT1i to hyperactivate CDK1 in the S-phase of *CCNE1* amplified cells and drive catastrophic amounts of DNA damage by forcing S-phase cells into mitosis. The differential mechanism of action between PKMYTi-ATRi and WEE1i-ATRi can potentially be attributed to the observations that WEE1i increases origin firing and DNA replication stress via activation of Cyclin E-CDK2 leading to greater reliance on ATR to stabilize and restrict replication fork progression^42,43^, whereas PKMYT1i has little effect on CDK2 activation^17^. Taken together this study indicates that CCNE1 amplified OVCA and EMCA cells are specifically vulnerable to CDK1 activation and strategies to increase CDK1 activation offer an attractive therapeutic avenue for this unmet need in the clinic. Preclinically, other combinations targeting CCNE1 overexpression include CDK2 inhibition (e.g dinaciclib) with AKT inhibition, CDK2/9 and PIK3CA inhibition, and WEE1 with PKMYT1 inhibition^44–47^.

There are several strategies targeting *CCNE1* amplification or CCNE1 overexpressing solid tumors that are under clinical development. Targeting WEE1 with ZN-c3, CHK1/2 with LY2606368, and CDK2 with INX-315, BLU-222, INCB123667, or ARTS-021 are all in early phase I/II monotherapy clinical trials (clinicaltrials.gov). Thus far, CDK2 inhibitors have largely failed in in clinical trials due to insufficient selectivity^10^. Clinical trials of combination regimens targeting CCNE1 are also in development. Given the results of this study showing that *CCNE1* amplification or overexpression represents a strong biomarker for sensitivity to low dose combination PKMYT1i (RP-6306) with ATRi (RP-3500), this combination has moved forward into the clinic as a phase 1 dose escalation study in advanced solid tumors with *CCNE1* amplification (NCT04855656; MYTHIC). PKMYT1i in combination with the chemotherapies gemcitabine and FOLFIRI is also being explored given preclinical data showing this combination is synergistic and similarly resulted in mitotic catastrophe (MAGNETIC and MINOTAUR: clinical trials.gov)^17^. Chemotherapy combinations with targeted agents such as older generation WEE1i have demonstrated activity but have been overall intolerable because of toxicity^48^.

Our data demonstrates that *CCNE1* gene amplification and overexpression are important biomarkers for sensitization to PKMYT1 combined with ATR inhibition. However, there is a clinical need to determine if *CCNE1* overexpression either by gene copy number (CN) or protein levels better correlates with response to agents targeting this oncogene. The optimal copy number threshold for *CCNE1* amplification, and the role of cyclin E protein levels as predictive biomarker of response is currently being investigated across preclinical and clinical studies.

In summary, we have identified a potential treatment option for an aggressive subset of OVCA and EMCA patients who have poor prognosis and limited treatment options. By exploiting oncogene-addicted cell-cycle checkpoints and DNA repair mechanisms with combination PKMYT1 with ATR inhibition, normal cells should be spared allowing lower dosing strategies thereby limiting toxicity. Translational endpoints in ongoing and future clinical trials with this drug combination and additional preclinical studies are crucial to define the optimal *CCNE1* CN (or protein) level to predict sensitivity to this drug combination.

## Online Methods

### Cell lines and primary cells

OVCAR3, FUOV1, KLE cell lines were purchased from ATCC (Manassas, Virginia); FUOV1 was obtained from Leibniz Institute DSMZ; OVCAR8 was obtained from NCI-DTP; Kuramochi, OVSAHO and OVKATE obtained from the Japanese Collection of Research Bioresources Cell Bank (JCRB). SNU685 from AcceGen Biotech (Fairview, NJ). CCNE1^Amp^ lines (copy number [CN] > 5) were: OVCAR3, FUOV1, COV318, KLE; CCNE1Gain (CN 2–5): OVCAR8, OVSAHO, Kuramochi; CCNE1 copy neutral (CCNE1Low): OVKATE, WO-20, SNU685. Ovarian cancer cell lines included: OVCAR3, FUOV1, COV318, OVCAR8, OVSAHO, WO-20, OVKATE. Endometrial cancer cell lines included: KLE, SNU685. OVCAR3, OVCAR8, Kuramochi, OVSAHO, OVKATE and SNU685 cells were maintained in RPMI 1640 media with 10% fetal bovine Serum (FBS; Thermo Fisher) and 1% penicillin/streptomycin (P/S; Thermo Fisher). FUOV1 and KLE cells were cultured in Dulbecco’s Modified Eagle’s Medium (DMEM)/F12 media with 10% FBS and 1% P/S. RPE1-hTERT *p53*^*−/−*^ Cas9, RPE1-hTERT *p53*^*−/−*^ Cas9 *BRCA1*^*−/−* 49^, RPE1-hTERT *p53*^*−/−*^ Cas9 *ATM*^*−/−*29^ and RPE1-hTERT *p53*^*−/−*^ Cas9 CCNE1 overexpressing cells^17^ were grown in DMEM (Life technologies # 11965-092) with 10% FBS (Wisent #080150) and 1% Pen/Strep (Wisent #450-201-EL). FT282-hTERT *p53R*^*175H*^ WT (empty vector) and *CCNE1* overexpressing cell lines were obtained from Ronny Drapkin^13^ and cultured in DMEM: F-12(1:1) (Life technologies # 11330-032) with 5% FBS, 1% UltroserG (Pall Life Sciences #15950-017) and 1% Pen/Strep.

The WO-20 primary ovarian cancer tumor cultures were generated in Simpkins laboratory as previous^11^. WO-20 CCNE1 inducible and SNU685 CCNE inducible cells were established by lentivirus stable infection^11^. Cell lines were authenticated by short tandem repeat (STR) analysis at the Oncogenomics Core at Wistar Institute and confirmed mycoplasma negative by end-point PCR at the Cell Center Service at the University of Pennsylvania.

### PDX Studies

NSG mice (NOD/SCID IL2Rγ−/−) were purchased from the Stem Cell and Xenograft Core (SCXC) at the University of Pennsylvania (UPENN, Philadelphia, PA). All mice experiments were performed in adherence to the policies of NIH Guide for the Care and Use of Laboratory Animals and approved by the Institutional Animal Care and Use Committee (IACUC). Patient tumors were obtained from ovarian cancer debulking surgeries conducted at the Hospital of the UPENN and Pennsylvania Hospital (IRB# 702679).

Orthotopic PDX models were generated by surgically engrafting of patient tumor chunks (3–4 pieces, 2mm3 each) to the ovary/oviduct of five-eight week old female mice as previously described^11,50^. The harvested PDX tumors were either retransplanted to NSG mice for further expansion or cryopreserved for future use.

We used in this study two high grade serous ovarian cancer (HGSOC, WO-19, WO-77) PDX models and one endometrial cancer (EMCA, WU-115) PDX model. For preclinical trials, cryopreserved PDX tumor tissue was thawed and transplanted. After tumors were palpable (~3–4mm), tumor volume was measured weekly by ultrasound (SonoSite Edge II Ultrasound System) by a trained sonographer. Tumor volume criteria for randomization to treatment arms was 50–100 mm3. Animals were randomized in a blinded manner into 6 treatment groups: vehicle (0.5% methyl cellulose); RP6306 (10mgkg, BID/day 1–5 weekly), RP6306 (5mg/kg, BID/day 1–5 weekly), RP3500 (5mg/kg, QD/day 1–3 weekly), combination RP6306 (10mg/kg, BID/day 1–5) + RP3500 (5mg/kg, QD/day 1–3), combination RP6306 (5mg/kg, BID/day 1–5) + RP3500 (5mg/kg, QD/day 1–3). Drugs were dosed by oral gavage. In all the models, percentage change in body weight during treatment was used as a marker for toxicity and dose level adjustments. Significant treatment toxicity was defined as a 15% drop in body weight and the mice require treatment reduction at 25% dose and supplements supportive. For mice with 20% drop in body weight, treatment was stopped and supportive measures (i.e., food supplement and subcutaneous fluid) were provided. The body weights, and condition scores of mice were monitored and recorded weekly. Once improved, treatment was restarted with a 25% dose reduction. If body weight was not regained after one week, animal was sacrificed in accordance with the Institutional Animal Care and Use Committee (IACUC) protocols. Trial endpoints were defined as tumor volume > 1000 mm3 for all the orthotopic PDX models. In the WU-115 model, two mice in the RP-6306 (10 mg/kg) + RP-3500 group were monitored without detectable tumors starting at week 38. Their tumors were palpated weekly but not body weight not measured, which leads to unchanged body weight in from week 38 to week 56 Fig. 3A.

### Cell line-derived xenografts

Animals were housed and experiments were performed at Repare Therapeutics (Admare Bioinnovations Montreal site, St-Laurent, Canada), which is a CCAC (Canadian Council on Animal Care) accredited vivarium. Studies were conducted under a protocol approved by the Admare Animal Care Committee (AACC). Mice were inspected upon arrival and group-housed (3–5 per cage) in individual HEPA ventilated autoclaved cages (Blue Line, Techniplast, Buguggiate, Italy) in a temperature-controlled environment (22±1.5°C, 30–80 % relative humidity, 12-h light/dark). Animals were provided with autoclaved corncob bedding, irradiated food (Harlan Teklad, Montreal, Canada) and filtered, autoclaved water ad libitum. They were also provided with nesting material and a plastic shelter as enrichment. Fresh bedding, nesting material and water was replenished/replaced on a weekly basis. Mice were acclimatized in the animal facility for at least 5 days prior to use and were identified with indelible ink. Experiments were performed during the light phase of the cycle.

OVCAR3 cells were implanted at 5×10^6^ cells per mouse into the right flanks of female SCID-beige mice respectively (5–7 weeks old; Charles River), in 1:1 Matrigel:media (ECM gel Sigma, cat# 1270; media Corning RPMI 1640 cat #10-41-CM). When tumors reached the average target size of ~150 mm^3^ (between ~100 and 200 mm^3^), (n=8) mice were randomized to treatment groups according to tumor volume and body weight using the “stratified” method in Studylogv4.4 software, and treatment with lunresertib and camonsertib was initiated. Lunresertib was formulated in 0.5% methylcellulose and orally administered twice daily (BID, 0–8h) for cycles of 3 days on/4 days off, for 28 days (4 cycles). Camonsertib was formulated in 0.5% methylcellulose and 0.02% SLS (pH 6.00) and orally administered once daily (QD) for cycles of 3 days on/4 days off, for 28 days (4 cycles). Statistical significance relative to vehicle control and other test groups was established by one-way Brown Forsyth and Welch ANOVA tests followed by unpaired t with Welch’s correction, with individual variances computed for each comparison for multiple groups and unpaired t-test for two group comparisons (GraphPad Prism v9.0).

### Blood and tumor tissue collection

Under isoflurane anesthesia, whole blood was collected by cardiac puncture and transferred to tubes containing 0.1 M citric acid (3:1 citric acid:blood) and stored at −20°C for LC-MS/MS analysis. Tumors were removed from mice flanks and cleared of surrounding mouse stroma. Tumor pieces between 50 mg and 100 mg were collected in a pre-weighed pre-filled bead mill tube (Fisher Scientific, Cat# 15-340-154) and then flash-frozen in liquid nitrogen. Other tumor fragments from vehicle- and compound-treated mice were placed in 10% neutral buffered formalin (NBF) within 2–3 minutes of surgical excision, fixed in NBF for 24 hours at room temperature and embedded in paraffin.

### RP-6306 and RP-3500 Quantitation by LC-MS-MS

The extraction of whole blood samples was performed by protein precipitation using four volumes of acetonitrile. The sample extracts were analyzed using a Transcend LX2 / Ultimate 3000 liquid chromatography system coupled to a Thermo Altis triple quadrupole electrospray mass spectrometer (Thermo Fisher Scientific) operated in positive mode. Separations were performed using a 2 × 50mm, 2.8µm Pursuit XRS C8 HPLC column (Agilent). A reversed-phase linear gradient of water + 0.1% formic acid and 1:1 acetonitrile:MeOH was used to elute RP-6306, RP-3500 and the internal standards. Samples were quantified against a 12-point linear standard curve and 5 levels of quality control samples. Whole blood concentrations of RP-6306 and RP-3500 were converted to free unbound plasma concentrations using an in vitro derived blood / plasma ratio = 1.2 and fraction unbound (f_u_) plasma = 0.185 for RP-6306 and blood / plasma ratio = 0.613 and fraction unbound (fu) plasma = 0.00665 for RP-3500 from the CD-1 mouse strain.

### Immunohistochemistry

Histology in Fig 4F, Fig. 6E and Fig. S9C was performed by HistoWiz Inc. Briefly, the formalin-fixed tissues were dehydrated through a 20%, 80%, 95% and 100 % ethanol series, cleaned in Histoclear, embedded in paraffin then sectioned at 4 μm. Immunohistochemistry for γH2AX and CDK1pT14 were performed on a Bond Rx autostainer (Leica Biosystems) with heat antigen retrieval. Bond polymer refine detection (Leica Biosystems) was used according to manufacturer’s protocol. After staining, sections were dehydrated and film coverslipped using a TissueTek-Prisma and Coverslipper (Sakura). Whole slide scanning (40x) was performed on an Aperio AT2 (Leica Biosystems). Image quantification analysis was performed using HALO. H-score is given by the formula: H-score = (1x % weak positive cells) + (2x % moderate positive cells) + (3x % strong positive cells). Histology in Fig. S3B was performed by Mosaic Laboratories (A CellCarta Company). Immunohistochemistry for Cyclin E1 (rabbit clone EP126) was performed in according to Mosaic’s standard operating procedures. This assay was designed and validated to be a laboratory-developed test. After heat-induced epitope retrieval, staining was performed on a Bond-RX autostainer (Leica Biosystems) and visualized with DAB chromogen. Slides were then removed from the instrument dehydrated, cleared and coverslipped. Stained slides were evaluated by a board-certified pathologist on a semi-quantitative scale, and the percentage of tumor cells staining at each of the following four levels was recorded: 0 (no staining), 1+ (weak staining), 2+ (moderate staining) and 3+ (strong staining). H-Score was calculated based on the summation of the product of percent of cells stained at each staining intensity using the following equation: (3 x % cells staining at 3+) + (2 x % cells staining at 2+) + (1 x % cells staining at 1+).

### Establishment and characterization of primary ovarian cancer organoids

Tumor tissue samples were set on a sterile petri dish and necrotic tissue were removed. The tumor was dissected to a 5 mm square under sterile conditions and washed with HBSS. Cleaned tissues were placed in a new petri dish and then minced. The minced tissues were mixed with enzymatic digestion buffer containing HBSS, collagenase 4 (1mg/ml), Rock Inhibitor (Y-27632). The mixture was placed in a 50 mL tube in a water bath at 37 °C for 15 min. The mixture was collected and dripped through a cell strainer on a new 50 mL tube to remove any residual tissue. The suspension was centrifuged at 300×g for 5 min at room temperature, the supernatant was removed. In case of a visible red pellet, erythrocytes were lysed in RBC Lysis buffer for 5 min at room temperature followed by two wash steps with 10 mL of HBSS and centrifugation at 300×g for 5 min. The cell pellet was suspended in Matrigel, and 50 μL drops of matrix cell suspension were allowed to solidify on a pre-warmed 6-well plate at 37 °C for 15 min. On stabilization of the Matrigel, we added the organoid medium cocktail^51^. The culture media is Advanced DMEM/F12 (Thermo Fisher Scientific, Cat#12634010), containing 2mM Glutamax (Thermo Fisher Scientific, Cat# 35050061), 10mM HEPES(Sigma-Aldrich, Cat# H0887-100ML), 100unit Pen Strep (Gibco, Cat# 15140-122), 100ng/ml Noggin (PeproTech, Cat#120-10C-100ug),100ng/ml R-Spondin-1 (PeproTech, Cat# 120-38-100ug), 1X B27(Thermo Scientific, Cat#17504001), 1.25mM N-Ace-L-Cys (Sigma, Cat#A9165-5G), 100 ug/ml Primocin (Invivogen, Cat# ant-pm-1), 10mM Nicotinamids (Sigma, Cat#N0636-500G), 500nM A83-01 (Tocris, Cat#2939), 10ng/ml FGF10 (PeproTech, Cat#100-26-50ug), 10ng/ml FGF2 (PeproTech, Cat#100-18B), 10uM SB202190 (Sigma-Aldrich, Cat#S7076-5MG),1uM PGE2(Tocris, Cat#2296-10mg), and 50ng/ml EGF(PeproTech, Cat#AF-100-15-500ug). The medium was changed every 3–4 days, and the organoids were passaged at a 1:2–3 dilution every 2–4 weeks. For passaging, organoids were mechanically and enzymatically dissociated into small clusters. Matrigel embedded organoids were suspended in Cell Recovery Solution (Corning, 500 µL/well). The organoid suspension was occasionally mixed with gentle pipetting for 30 min on ice to completely solubilize the Matrigel. The tube was then placed on ice to precipitate the organoids. The supernatant was removed, and organoids were washed with 1ml cold PBS. The organoid was suspended in Matrigel, and plated on 6-well plate.

After established, organoids were processed for paraffin sectioning using standard protocols for characterization. Matrigel embedded organoids were suspended in Cell Recovery Solution (Corning, 500 µL/well). The organoid suspension was occasionally mixed with gentle pipetting for 30 min on ice to completely solubilize the Matrigel. The tube was then placed on ice to precipitate the organoids. The supernatant was removed, and organoids were washed with cold PBS. Organoids were fixed with 4% paraformaldehyde (PFA) for 20 min at room temperature, and solidified using histogel before embedding in paraffin. 5 μm sections were stained with hematoxylin–eosin (H&E) and Antibodies (p53, PAX8, CK7).

### *In vitro* cell viability assay (MTT assay)

Cells were seeded into 96-well plates at 5000 cells/well. Cells were treated with control (DMSO), RP-6306, RP-3500 or combination at indicated concentrations for 5 days. Drugs were clinical grade and obtained from Repare Therapeutics. At the end of the treatment period, an MTT colorimetric assay was performed to detect the cell viability. Cells were incubated with 10 mL of MTT at 5 mg/ml (Sigma Chemical Co., St Louis, MO) for 4 h at 37 degree. The supernatant was removed and 100 mL DMSO (Fisher Scientific, Hampton, NH) was used to dissolve the MTT formazan. Absorbance was measured in a microplate reader at a wavelength of 570 nm. Relative cell viability was calculated, with the non-treatment group as a control.

### Colony formation assay

For colony formation assay, cells were plated onto 24-well at 5000 cells/well and cultured overnight in triplicate. They were then treated with DMSO vehicle, RP-6306, RP-3500 or combination as indicated every 3 days for a total of 10 days. Cells were then fixed and stained with 0.1% Crystal violet in 20% methanol solution. The plates were washed, air-dried, scanned, and quantified in ImageJ (National Institutes of Health, Bethesda, MD).

### Western blotting assay

Cells were treated and collected at indicated time, then washed and incubated with Laemmli Sample Buffer (4% SDS, 20% Glycerol, 0.12M Tris-HCl at pH 6.8 in distilled water) containing a protease and phosphatase inhibitor cocktail (EMD Millipore, Billerica, MA). After measured protein concentration with BCA kit (BioRad, Hercules, CA), whole cell lysates (15 mg) were separated on reducing 4%–15% SDS-PAGE gels, electrotransferred to PVDF membrane (Bio-Rad, Hercules, CA), blocked with 5% BSA (ThermoFisher) in 1x Tris-buffered saline (ThermoFisher) with 0.1% Tween20 (ThermoFisher) (1x TBST), and immunoblotted with respective primary antibodies including anti-pCHK1(S345) (Cell Signaling Technology, Danvers, MA, cat.#2348), anti-CHK1 (Cell Signaling Technology, cat.#2360), anti-CHK1 (Santa Cruz G-4 sc-8408), anti-CHK1-phosphoS345 (Bethyl, cat#2348), anti-Alpha Actinin (Millipore Sigma 05-384), anti-CDC25B (Thermofisher OTI6H9 TA8-12352), anti-CDC25B-phosphoS151 (Thermofisher PA5-104568), anti-pCDK1(T14) (Abcam, Cambridge, UK, cat.# ab58509), anti-CDK1(Cell Signaling Technology, cat.#9116), anti-γH2AX(Cell Signaling Technology, cat.#9178), anti-cleaved caspase 3(Cell Signaling Technology, cat.#9664), Cyclin E1(Cell Signaling Technology, cat.#4129), anti-Actin(Cell Signaling Technology, cat.#3700), anti-ATM (Cell Signaling Technology,cat#2873), anti-BRCA1 (Dan Durocher, University of Toronto). After that, membranes were washed and blotted with species-appropriate horseradish peroxidase conjugated anti-rabbit (catalog 7074, Cell Signaling Tech), anti-mouse (catalog 7076, Cell Signaling Tech) secondary antibodies or anti-mouse Irdye 800CW (LiCOR 926-32210), anti-mouse Irdye 680CW (LiCOR 926-68072), anti-rabbit Irdye 800CW (LiCOR 925-32213) and anti-rabbit Irdye 680CW (LiCOR 926-68073) in 5% BSA in 1x TBST for 1 h, followed by chemiluminescent substrate (Thermo Scientific, Rockford, IL) incubation and film development. Actin or Actinin was used as loading control for whole cell.

### Flow cytometry detection of intracellular proteins

Cells were seeded in triplicate and then treated with RP-6306, RP-3500 or combination for indicated time. Cells were then trypsinized, fixed washed and incubated with blocking buffer. Cells were then stained with the following primary antibodies diluted in blocking buffer at 1:300: gH2AX (Cell Signaling Technology, cat# 9718), pRPA32 (S33, Bethyl Laboratories, cat#A300-246A) or phospho-histone H3 (Ser10, Cell Signaling Technology, cat# 53348). The cells were washed, and incubated with secondary antibody goat anti-Rabbit IgG (H+L), Alexa Fluor 647 (ThermoFisher Scientific) for 30 min. The cells were then incubated with 50 mg/mL propidium iodide (Sigma-Aldrich) and subjected to flow cytometry acquisition on BD LSRII (BD Biosciences) and data analysis with FlowJo (Tree Star, Inc., Ashland, OR).

### Apoptosis analysis

Cells were plated, incubated overnight, and treated with DMSO vehicle, 250 nM RP-6306, 50nM RP-3500, or combination for 72 h. Apoptosis assay was performed with eBioscience Annexin V Apoptosis Detection Kit APC (Invitrogen, 88-8007-74), according to the manufacturer’s instruction. Annexin V-APC and propidium iodide labeled cells were detected by BD Accuri C6 Cytometer (BD Biosciences, San Jose, CA). The acquired data was analyzed with FlowJo (Tree Star, Inc., Ashland, OR).

### High content imaging and quantitative image-based cytometry (QIBC)

High-throughput analysis of nuclear γ-H2AX, Histone H3-phosphoS10 and Cyclin B1-phosphoS126 cells was done as preciously described^17^. Briefly, cells were seeded in 96-well plates (3000 cells/well for FT282-hTERT *p53*^*R175H*^) and cultured for up to 24–48 h depending on the experiment. Prior to harvesting, cells were pulsed with 20 μM EdU (5-ethynyl-2-deoxyuridine, Life Technologies #A10044) for 30 min followed by addition of paraformaldehyde (PFA) in PBS to a final concentration of 4% and incubated for 15 min at room temperature (RT). Cells were then rinsed with PBS and permeabilized using 0.3% Triton X-100/ PBS for 30 min. For chromatin-bound γH2AX and RPA measurements, cells were pre-extracted for 15 min on ice with CSK buffer (300 mM sucrose, 100 mM NaCl, 3 mM MgCl_2_, 10 mM PIPES pH 7.0, 0.5% v/v Triton-X 100) before PFA fixation. Cells were rinsed with PBS and incubated with EdU staining buffer (150 mM Tris-Cl pH 8.8, 1mM CuSO_4_, 100 mM ascorbic acid and 10 μM AlexaFluor 488 azide (Life Technologies, #A20012) for 30 min. Cells were washed with PBS and incubated in blocking buffer (10% goat serum (Sigma #G6767), 0.5% NP-40 (Sigma-Aldrich, #I3021), 5% w/v Saponin (Sigma-Aldrich, #84510), diluted in PBS) for 30 min. Fresh blocking buffer containing primary antibodies was added for 2 h. Primary antibodies including histone H2A.X (phospho-S139, Millipore Sigma #05-636, 1:500 IF), RPA32 (Abcam ab2175, 1:500 IF), Histone H3-phosphoS10 (Cell Signaling Technology #9706, 1:500 IF), Cyclin B1-phosphoS126 (Abcam ab55184, 1:500 IF). Cells were rinsed three times with PBS and then blocking buffer, with secondary antibodies including AlexaFluor488 goat anti-mouse IgG (Thermo Fisher Scientific A11029, 1:1000), AlexaFluor647 goat anti-rabbit IgG (Thermo Fisher Scientific A21244, 1:1000) and AlexaFluor555 goat anti-mouse IgG (Thermo Fisher Scientific A28180, 1:1000). Then 0.4 μg/mL DAPI (4,6-diamidino-2-phenylindole, Sigma-Aldrich, #D9542) was added for 1 h. After rinsing with PBS, immunocomplexes were fixed again using 4% PFA/PBS for 5 min. Cells were rinsed with PBS, wells were filled with 200 μl PBS and images were acquired at the Network Biology Collaborative Centre (LTRI) on an InCell Analyzer 6000 automated microscope (GE Life Sciences) with a 20X objective. Image analysis was performed using Cellprofiler 3.1.9^52^ and RStudio v1.2.5019 in a similar manner as previously described^17^.

### Statistical Analysis

In vitro studies were performed using at least 3 biological replicates per sample and 3 independent experiments. Two-tailed unpaired t tests were used when comparing two groups. One-way ANOVA followed by Tukey’s post hoc comparison was performed for multiple group comparisons. p < 0.05 was considered statistically significant. Drug interaction between RP-6306 and RP-3500 was analyzed using the coefficient of drug interaction (CDI)^53^. CDI = AB/(AxB); AB is the ratio of two-drug combination group to control, and A or B is ratio of a single drug to control. CDI < 1 indicates synergism, CDI < 0.7 indicates significant synergism, CDI = 1 indicates additivity, and CDI > 1 indicates antagonism. GraphPad Prism (Graphpad Software version 10.0.2, San Diego CA) was used for statistical analyses.

For statistical power for *in vivo* studies, there were 4–10 mice/arm. Weekly ultrasound measurements, weights, and condition scores were obtained. Longitudinal analysis of tumor growth was carried out by linear mixed-effect modeling with type II ANOVA and pairwise comparisons across groups on log pre-processed tumor sizes using the TumGrowth web tool (https://kroemerlab.shinyapps.io/TumGrowth/)^54^ Natural log transformed tumor volume was used to better satisfy normal distribution. Survival data was analyzed by Mantel-Cox log rank test. Survival data was analyzed by Mantel-Cox log rank test.

## Supplementary Material

Supplement 1

## Data Availability

All the other data supporting the findings of this study are available within the article and its supplementary information files and from the corresponding author upon reasonable request.
