# Supplementary material for: Targeting CCNE1 amplified ovarian and endometrial cancers by combined inhibition of PKMYT1 and ATR
Source: Res Sq. 2024 Feb 16:rs.3.rs-3854682. Preprint. [Version 1] doi: 10.21203/rs.3.rs-3854682/v1 (PMC10896384; doi:10.21203/rs.3.rs-3854682/v1)
Supplement: Supplement 1 [file NIHPPrs3854682v1-supplement-1.pdf]

Supplementary Files

This is a list of supplementary files associated with this preprint. Click to download.

- SupplementaryFigures20240111Final.pdf
- SupplementoryFigureLegends.pdf
